# Accuracy of the third molar maturity index (I3M) for diagnosing the legal majority of young southern Brazilians

**DOI:** 10.4317/jced.61423

**Published:** 2024-04-01

**Authors:** Vanessa-Koltermann Sartori, Ademir-Franco-do Rosário Júnior, Pedro-Henrique Corazza, Yuri-Dall Bello, Rizky-Merdietio Boedi, Felipe-Gomes Dallepiane, Letícia-Copatti Dogenski, Maria-Salete-Sandini Linden, Micheline-Sandini Trentin, João-Paulo De Carli

**Affiliations:** 1 Post-Graduation Program in Dentistry at the University of Passo Fundo, Passo Fundo, Rio Grande do Sul, Brazil; 2 Post-Graduation Program in Dentistry at the São Leopoldo Mandic Research Center, Campinas, São Paulo, Brazil; 3 Odontologi Forensik dan Medikolegal, Fakultas Kedokteran, Universitas Diponegoro; 4 Post-Graduation Program in Dentistry at the Federal University of Santa Catarina, Florianópolis, Santa Catarina, Brazil

## Abstract

**Background:**

The numerous techniques for identifying adulthood require research testing the accuracy of each method in different populations. This study verified the accuracy of the third molar maturity index (I3M) proposed by Cameriere et al. (2008) for diagnosing the age of majority in a southern Brazilian population sample.

**Material and Methods:**

Panoramic radiographs of patients with dental element 38 treated at the School of Dentistry of the University of Passo Fundo (UPF), RS, Brazil, were analyzed. The patients were separated into age groups between 15.00 and 22.99 years. The Cameriere (2008) method was applied to each radiograph. The study sample comprised 671 individuals, with 385 women (mean age 19.67 ± 2.05) and 286 men (mean age 19.5 ± 2.11).

**Results:**

The original cut-off value of I3M≤0.08 classified individuals younger and older than 18 years. ROC curve plotting resulted in an overall accuracy of 0.69 and 0.84 for women and men, respectively. The most favorable cut-off value for southern Brazilian men was 0.06, and women showed better results with an I3M adjusted to 0.13. The new cut-off values produced an accuracy of 0.78 for women and 0.84 for men. The original cut-off point to the I3M (0.08) was not the most appropriate for the studied sample.

**Conclusions:**

Thus, index adjustments to 0.13 for women and 0.06 for men may improve method performance among southern Brazilian individuals.

** Key words:**Molar, third, radiography, panoramic, forensic dentistry, age groups, imputability.

## Introduction

In Brazilian law, the age of 18 is a milestone for attaining civil and criminal majority, and establishing this age is relevant for civil rights acquisition and sentence application due to criminal practice. Diagnosing the age of majority becomes vital in the civil sphere, and it is commonly used in adoption cases (1), refugee asylum requests (2), and cases of missing or false civil records (3), considering that Brazilian legislation establishes civil rights and duties according to age groups. Therefore, forensic dentistry detects the age of majority, and it is crucial for identifying individuals in mass disasters, such as accidents and earthquakes, and criminal cases, such as homicides, rapes, and suicides, even with limited availability of human remains or samples (4).

Detecting the age of majority for forensic purposes is highly relevant because of legal implications (5) and migration issues (6). Therefore, the techniques must have specific standards for each population, seeking higher precision (7), particularly when estimating the ages of people from diverse backgrounds (8).

Bone and/or dental development supports adulthood detection and is researched to develop increasingly precise scientific methods (9). Dental element formation has mineralization stages according to human growth. Such stages accurately diagnose age in childhood when teeth are in complete development. However, this accuracy tends to decrease as tooth mineralization finishes. Thus, third molars may help identify adulthood in the youth stage, especially for individuals aged around 18 years (10).

The method by Cameriere *et al*. (2008) analyzes the root apices of third lower left molars, determining the third molar maturity index (I3M) (11). In Brazil, Nóbrega *et al*. evaluated the applicability of the I3M (12) originally proposed by Cameriere *et al*. (11) to estimate the age of majority in a population sample aged 14 to 23 years in northeast Brazil, using 394 panoramic radiographs. The age classification was correct in 80.2% of the sample without accuracy differences between the sexes. The original cut-off point proposed for the I3M (0.08) accurately distinguished the age of adolescents and young adults in the studied population (overall accuracy of 80.8%). However, Goetten *et al*. 2022 analyzed 1,070 panoramic radiographs using the I3M in northern Brazilian individuals between 16 and 22 years old, concluding that the best I3M cut-off point for men remained at 0.08, and women showed better results (98.5% accuracy) with an adjustment to 0.12 (13). Therefore, the methodologies must be applied to different populations because of potential differences in technique accuracy according to the sampled group (14).

Therefore, the present study is justified because the Cameriere (2008) method (11) has not been used to diagnose the age of majority in the southern Brazilian population, and the accuracy of this tool must be verified. This analysis seeks to confirm the hypothesis that a 0.08 cut-off point for the I3M proposed by Cameriere (2008) (11) is the most appropriate for detecting adulthood in a southern Brazilian population.

## Material and Methods

-Ethical aspects and sample

The Research Ethics Committee of the University of Passo Fundo approved this study (opinion number 3,688,526). It is an analytical cross-sectional observational radiographic study of all panoramic radiographs selected from the dental records of patients who attended the School of Dentistry of the University of Passo Fundo, RS, Brazil, seeking dental treatment from 2016 to 2022. A retrospective survey of the following data was also performed in the dental records: date of birth, sex, and image acquisition date. Patients were separated into age groups between 15.00 and 22.99 years. The Cameriere (2008) method (11) was applied to each radiograph to verify the I3M of the lower left third molar (tooth 38), considering I3M≤0.08 for individuals aged 18 years or older.

An Eagle Digital™ device (Dabi Atlante, Ribeirão Preto, SP, Brazil) captured the radiographs. The sample was selected by convenience, and all radiographic images were exported to JPEG file format. Next, they were saved in high resolution on a computer, organized into folders, and exported to Adobe™ Photoshop™ CC (PS™CC) software. Then, the image file was opened and enlarged, and when necessary, the brightness, contrast, and zoom were adjusted for better visualization. Before analyzing the radiographs, all indications of patients’ sex and age were removed, and the images were filed under new registration numbers.

-Application of the Cameriere (2008) method (I3M)

**Figure 1:**
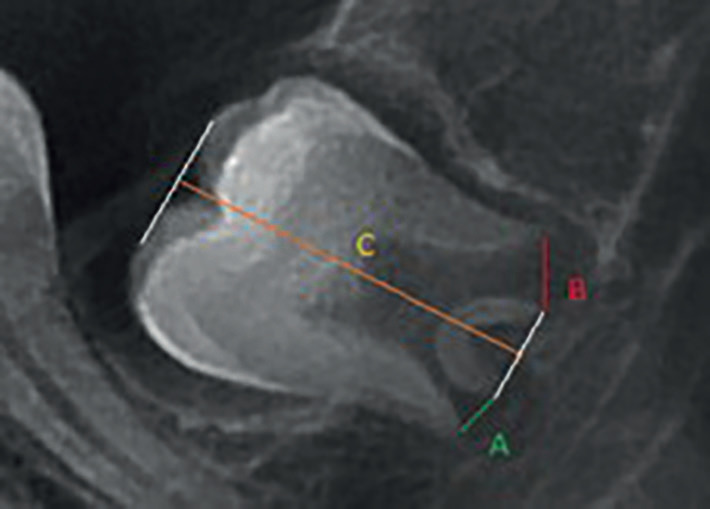
Illustrative image of measurements for applying the Cameriere (2008) method. Note the used measurements: the sum of distances between the internal walls of the two open apices (A + B) divided by tooth length (C).

The Cameriere (2008) method was applied to the sample based on the analysis of lower left third molar apices – tooth 38 (LL3M), determining the third molar maturity index (I3M). Thus, if the LL3M presented complete root development (completely closed apex), the I3M was zero. Otherwise, I3M was calculated by adding the distances between the internal walls of the two open apices (A + B) and dividing by tooth length (C), as seen in Figure 1. Cameriere (2008) (11) stated that the I3M value limit is 0.08, i.e., individuals with I3M≤0.08 are 18 years or older, and those with I3M>0.08 are younger than 18 years.

A software image file (<File-Open>) provided the measurements. The ruler in the PS™CC toolbar measured the internal side of the open apex of LL3M mesial and distal roots. The pen tool determined tooth length by drawing two lines: an upper one tangent to the two uppermost cusps and a lower one tangent to the lower limit of the walls forming the furcation, mesial, and distal roots. Next, the ruler tool measured the length limited to the midpoints of the drawn lines. In the absence of furcation, the distance between the inner side of the outer walls of mesial and distal roots was measured, also serving as a basis for determining length.

-Inclusion and exclusion criteria

The study included only panoramic X-rays of patients between 15.00 and 22.99 years old who had undergone treatment at the School of Dentistry of the University of Passo Fundo, RS, Brazil, between 2016 and 2022 and who had dental element 38 favorably positioned. The study excluded low-quality panoramic radiographs, including image acquisition or processing errors, evident bone injuries, developmental disorders, and missing information about sex, date of birth, or image acquisition date.

-Statistical analysis

Epidemiological and I3M data were tabulated in a Microsoft Excel™ spreadsheet. Two examiners underwent training on the Cameriere (2008) technique according to methodological guidelines. Subsequently, Observer 1 (V.K.S.) evaluated 100% of the sample for I3M, and Observer 2 (A.F.R.J.) evaluated 10%. Intraobserver (ICC 0.92) and interobserver (ICC 0.85) agreements (ICC - intraclass correlation coefficient) were tested. The two assessments from Observer 1 were 45 days apart. The panoramic radiographs corresponding to 10% of the sample evaluated by Observer 2 were selected by simple random sampling, covering all ages of final sample patients.

-Comparison between chronological age and majority diagnosis

This study used the primary data extracted from 671 panoramic radiographs related to a continuous quantitative exposure variable (I3M), with age as the outcome variable. The sample consisted of 385 women (mean age 19.67 ± 2.05) and 286 men (mean age 19.5 ± 2.11), showing an age homogeneity among the analyzed subjects. The random sample comprised individuals between 15 and 22.99 years old and was stratified into groups, as seen in Table 1.

**Table 1:**
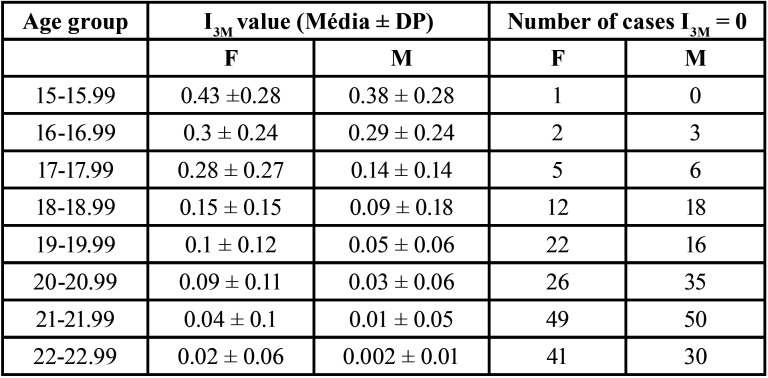
Distribution of I3M values in the studied population.

The RStudio program, version 4.2.1 (R Core Team, R Foundation for Statistical Computing, Vienna, Austria) analyzed the data. A logistic regression model calculated the probability of a legal majority in individuals, using I3M and sex as independent variables and chronological age as the dependent variable. Cut-off assessments tested three values - 0.08, 0.12, and a specific number for the studied population.

The following performance variables investigated each cut-off point: sensitivity (SEN), specificity (SPE), accuracy (ACC), and area under the curve (AUC). The value for the studied population was obtained by optimizing the cut-off point using the Youden index method (J), where J = SENS + SPEC - 1. All tests used the k-fold cross-validation method with five times and two repetitions.

## Results

Table 1 descriptively reports the I3M values. When analyzing them, it can be observed that the closer the I3M is to zero, the greater the chance of the individual being 18 years old or over. Furthermore, the older the subjects, the greater the number of individuals with I3M equal to zero, which means that they already have the apex of the lower left third molar closed, both in female and male individuals.

**Figure 2:**
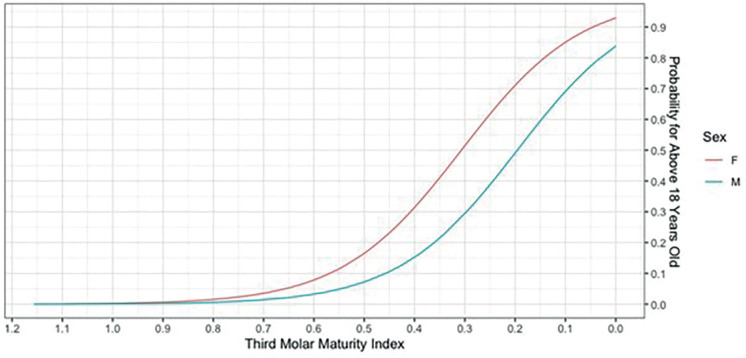
Graph representing the relationship between the I3M value (X-axis) and the probability of patients being 18 years or older (Y-axis).

In Table 2 it can be seen that the difference between I3M values and the age of the patients was -0.56 (*p*<0.05), revealing that there is a relationship between age and I3M. Thus, despite the expectation being moderate, we can conclude that the value of I3M is inversely proportional to age. In Figure 2 and Table 2 it can be seen that the correlation between I3M and age is greater for women compared to men.

**Table 2:**
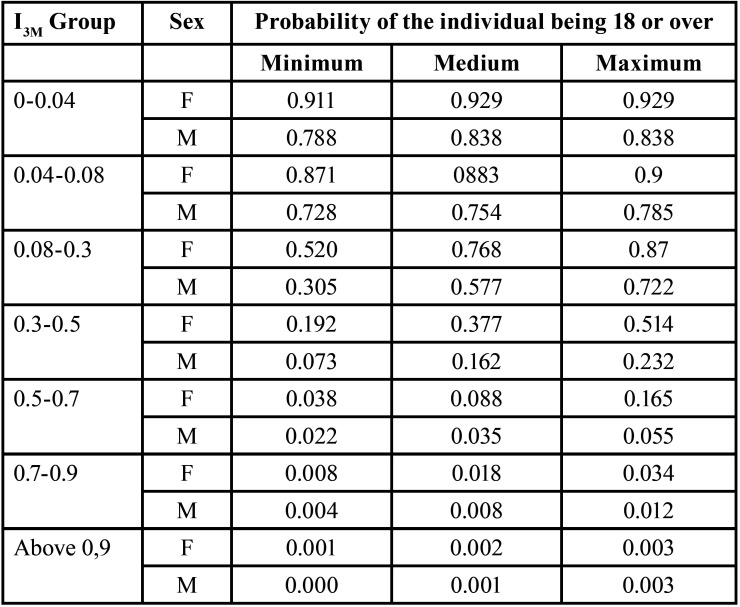
Correlation between I3M values and age stratified by patient sex.

**Figure 3:**
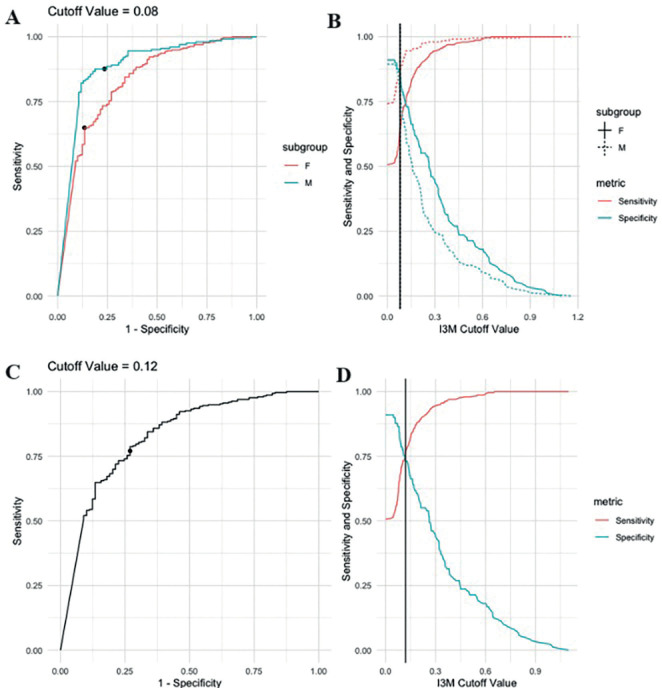
3AB (above-I3M 0.08) and 3CD (below-I3M 0.12) – Show respectively for males and females, and only females, the “sensitivity x specificity” and “I3M cut-off value x sensitivity” curves and specificity”.

The original cutoff point of 0.08 proposed by Cameriere *et al*. was tested. (2008), resulting in ACC of 0.69 and 0.84 for women and men, respectively (Table 3, Fig. 3). Furthermore, the cutoff value of 0.12, proposed by Goetten *et al*. (2022), was tested, which was shown to provide an improvement in general performance in female samples (ACC=0.76) (Table 3, Fig. 3).

**Table 3:**
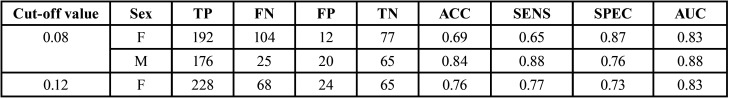
Accuracy (ACC), sensitivity (SENS), specificity (SPEC), and area under the curve (AUC) based on the analysis of the original I3M cut-off point proposed by Cameriere (2008) (11) in women and men (0. 08) and the cut-off point proposed by Goetten *et al*. (2022) (13) for women (0.12).

**Figure 4:**
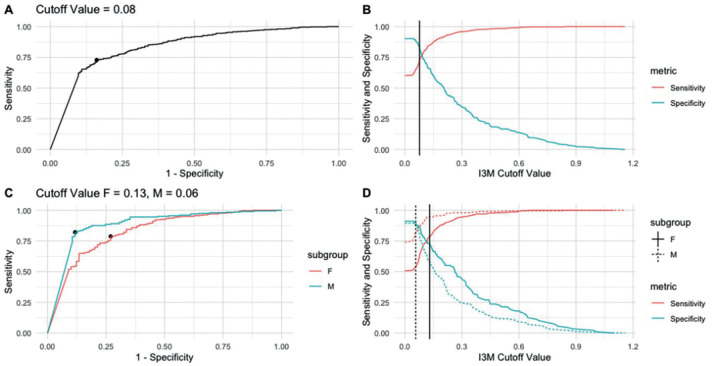
Comparison of curves obtained for the general population with a cut-off point of 0.08 (A, B) with those for women and men with cut-off points of 0.13 and 0.06, respectively (C, D).

A general specific cutoff value was also calculated for the studied population, which was 0.08 for both sexes, with ACC = 0.76 and J = 0.57. Additional calculations were made for each sex, resulting in a cutoff value of 0.13 for females (ACC = 0.78, J = 052) and 0.06 for males (ACC = 0.84, J = 0.70), as shown in Table 4 and Figure 4.

**Table 4:**
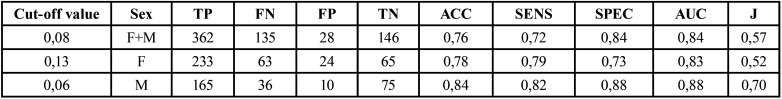
Values obtained with the general cutoff point for the studied population (0.08) and cutoff points established for females (0.13) and males (0.06).

## Discussion

The hypothesis of this study was partially rejected because, although being appropriate to the analyzed southern Brazilian population, the cut-off point proposed by Cameriere (2008) (11) (I3M≤0.08) was not the most appropriate, and it might be optimized differently for male and female fractions of the population. After establishing logistic regression models, the most favorable cut-off value for southern Brazilian men was 0.06 (sensitivity of 0.82; specificity of 0.88), and women showed the best results with an I3M adjustment to 0.13 (sensitivity of 0.79; specificity of 0.73). These new cut-off values promoted an accuracy of 0.78 for women and 0.84 for men.

That is probably because of ethnic differences between the analyzed populations, considering the original study by Cameriere (2008) (11) included Caucasian individuals, unlike the present research. Third-molar development is specific for each population, although there is not sufficient data to confirm the effects of ethnic origin on dental mineralization (15). Thus, the influence of ethnicity on age estimation must be investigated with a precise definition of populations and cultures (16), as third-molar mineralization does not occur at the same age in different populations (17,18).

Similar to this study, previous research has carefully adapted the I3M cut-off value for better applicability of the Cameriere (2008) method (11). Chu *et al*. (2018) studied 840 panoramic radiographs of northern Chinese individuals, finding that increasing the I3M value to 0.10 promoted higher accuracy in age discrimination for men and women. This threshold provided high sensitivity (0.929 and 0.809) and specificity (0.940 and 0.973) in men and women, respectively (19). In turn, Goetten *et al*. (2022) evaluated 1,070 panoramic radiographs of a northern Brazilian population between 16 and 22 years old, obtaining an accuracy of 73.1% for women and 80% for men. These authors adjusted the cut-off value to 0.12 for women and achieved 98.5% accuracy (13). Therefore, the authors encourage cut-off value adjustments to improve method performance among women.

Contrary to this investigation, previous studies indicated that a cut-off point of I3M≤0.08 effectively discriminated between adults and minors. Cavrić *et al*. (2016) exemplified this by using I3M to distinguish adults from individuals younger than 18 years among 1,294 black Africans between 13 and 23 years old from Gaborone, Botswana. Third-molar development did not show statistically significant differences between sexes (*p*>0.05). Method accuracy was 91% in men and 92% in women, sensitivity was 88% in men and 88% in women, and specificity was 94% in men and 96% in women. The I3M was highly accurate, representing a satisfactory alternative in legal and forensic practice to discriminate the age of majority in black African individuals (20). Similarly, Balla *et al*. (2017) (21) analyzed 216 panoramic radiographs of living individuals between 14 and 21 years old, observing high sensitivity (83.3% and 90.2%) and specificity (98.3% and 95.1%) for both sexes. The estimated post-test probability was 98.0% in women and 94.8% in men, and the specific cut-off value of I3M≤0.08 efficiently classified individuals around 18 years old in southern India (21).

Nóbrega *et al*. (2019) also found that the I3M cut-off point originally proposed by Cameriere (2008) (11) accurately distinguished the age of adolescents and young adults in a northeastern Brazilian population. These authors analyzed 394 panoramic radiographs of a sample aged 14 to 23 years. Sensitivity was 88.4%, specificity was 73.2%, accuracy (area under the ROC curve) was 80.8% (95% CI: 76.4–85.3%), and age classification was correct in 80.2% of the sample (12).

This research shows that the correlation between I3M values and patients’ age was -0.56 (*p*<0.05), demonstrating the absence of a relationship between age and I3M. Thus, despite the moderate correlation, the I3M value was inversely proportional to age, demonstrating consistency in the analyzed sample. That is because the older the individuals, the more sealed the third molar apices, resulting in a lower I3M. Nóbrega *et al*. (2019) studied 394 panoramic radiographs of northeastern Brazilians between 14 and 23 years old (12), and Thilak *et al*. (2021) analyzed 542 panoramic radiographs of Indian individuals between 14 and 24 years old (22), both concluding that I3M is inversely proportional to chronological age, corroborating our findings.

In the present study, it was possible to observe that the correlation between I3M and age is higher for women, with apical closure occurring first in the female gender. This means that as I3M decreases, the probability of female individuals being 18 years or older is higher than in males. This result differs from those of Nóbrega *et al*. (2019, who noted earlier apical closure in male individuals (12), and from the findings of Cameriere *et al*. (2008) and Thilak *et al*. (2021, which reported no differences in probability between the female and male samples regarding I3M and age (11,22). In relation to this aspect, our results coincide with those of Cavrić *et al*. (2016), who concluded that women are slightly faster in the development of permanent teeth when compared to men (20).

Studies performed in Brazil (12,13,23) have used the Cameriere (2008) method (11), but none involved the southern region. Brazil has continental dimensions, so the present research could not apply the technique to a significant sample of its population. However, a southern Brazilian sample confirmed the technique’s applicability. That is a limitation of our research and supports the need for further similar studies in different geographic regions of Brazil.

This study is highly relevant and applicable to dental forensics assisting legal claims (24,25). Therefore, additional studies with methods for identifying the age of majority through dental X-rays in Brazil are crucial to confirm our results.

## Conclusions

The I3M method effectively diagnosed the age of majority in a southern Brazilian population based on the analysis of panoramic radiographs. However, female and male individuals differed regarding the established cut-off point. The original cut-off value proposed for the I3M (0.08) was not the most appropriate for the sample. Thus, index adjustments to 0.13 for women and 0.06 for men may improve method performance among the analyzed population.
